# Attention-ProNet: A Prototype Network with Hybrid Attention Mechanisms Applied to Zero Calibration in Rapid Serial Visual Presentation-Based Brain–Computer Interface

**DOI:** 10.3390/bioengineering11040347

**Published:** 2024-04-02

**Authors:** Baiwen Zhang, Meng Xu, Yueqi Zhang, Sicheng Ye, Yuanfang Chen

**Affiliations:** 1 Institute of Information and Artificial Intelligence Technology, Beijing Academy of Science and Technology, Beijing 100089, China; zhangbaiwen0311@163.com; 2 Faculty of Information Technology, Beijing University of Technology, Beijing 100124, China; zslmdry@emails.bjut.edu.cn; 3 Intelligent Science and Technology, International College of Beijing University of Posts and Telecommunications, Beijing 100083, China; 2023213700@bupt.cn; 4 Beijing Institute of Mechanical Equipment, Beijing 100854, China

**Keywords:** Attention-ProNet, hybrid attention mechanism, rapid serial visual presentation (RSVP), zero-calibration (ZC), prototype networks

## Abstract

The rapid serial visual presentation-based brain–computer interface (RSVP-BCI) system achieves the recognition of target images by extracting event-related potential (ERP) features from electroencephalogram (EEG) signals and then building target classification models. Currently, how to reduce the training and calibration time for classification models across different subjects is a crucial issue in the practical application of RSVP. To address this issue, a zero-calibration (ZC) method termed Attention-ProNet, which involves meta-learning with a prototype network integrating multiple attention mechanisms, was proposed in this study. In particular, multiscale attention mechanisms were used for efficient EEG feature extraction. Furthermore, a hybrid attention mechanism was introduced to enhance model generalization, and attempts were made to incorporate suitable data augmentation and channel selection methods to develop an innovative and high-performance ZC RSVP-BCI decoding model algorithm. The experimental results demonstrated that our method achieved a balance accuracy (BA) of 86.33% in the decoding task for new subjects. Moreover, appropriate channel selection and data augmentation methods further enhanced the performance of the network by affording an additional 2.3% increase in BA. The model generated by the meta-learning prototype network Attention-ProNet, which incorporates multiple attention mechanisms, allows for the efficient and accurate decoding of new subjects without the need for recalibration or retraining.

## 1. Introduction

The rapid serial visual presentation-based brain–computer interface (RSVP-BCI) paradigm is based on the principle that the visual neural system elicits P300 event-related potentials in response to novel stimuli [1]. By presenting the experimental participants with an image sequence at a rate ranging from 2 to 20 Hz, it is possible to decode the target image on which the subjects are focusing from a massive pool of images using their electroencephalogram (EEG) patterns. Because this is an oddball event, the probability of the target image appearing is typically approximately 5–10% [2,3]. Initially used for psychological tests, such as short-term memory and attentional blinking, RSVP has been applied as a tool for target detection in various fields, including military target detection, topographic surveys, and medical image recognition. Following manual and machine searches, RSVP has evolved as a brain-inspired approach for target detection. This represents a novel paradigm in BCI that emerged after steady state visual evoked potential (SSVEP) and motor imagery [4,5].

Similarly to most BCI paradigms, RSVP also faces challenges, such as non-stationary EEG signals and significant individual differences, resulting in a less-than-ideal classification accuracy and poor generalization. Moreover, other issues, such as the cost of acquiring EEG signals, pose significant constraints on its practical application, especially in the field of object detection, where targets appear infrequently, leading to a scarcity of EEG samples [6]. These factors contribute to the challenge of models generated from training on a single subject, thus breaking free from the limitations of “one person, one application” or “one day, one application”. This greatly hinders the broader application of the RSVP paradigm.

To alleviate such limitations, researchers have tried using cross-subject learning approaches. Cross-subject learning means that a model is trained on one subject and directly applies it to new subjects. The purpose is to enhance the generalization capability of the brain–computer interface systems, enabling their widespread application across different populations without the need for personalized adjustments or training for each user. However, in the RSVP test environment, due to the need for handling scenarios where the target appears for the first time or in online settings, the RSVP paradigm must be capable of making predictions with zero-shot learning, a task referred to as zero-calibration (ZC) in BCI research. As a special case of transfer learning, the ZC method does not use test samples for model training, unlike traditional transfer learning, which requires using a small number of test samples for training. Therefore, it is a directive method that can address the issue of unlabeled data scarcity in RSVP paradigms.

The ZC method is a technology that was developed to address the issue of small sample sizes [7]. It aims to create a low-dimensional feature space, abstract brain signals into a universal representation, and calculate the mapping relationship between the source and target domains. Therefore, the system is better adapted to different brain signal patterns, i.e., ZC performed by learning the mapping between stimulus paradigms and EEG signals, which allows new EEG test data to be well classified in the target domain. Furthermore, it has been considered an effective approach for addressing generalization issues in BCI systems [8,9,10]. 

In some studies, ZC models have been explored for various BCIs. Currently, data-driven ZC EEG decoding includes methods based on ensemble integration using existing classifiers, pooled methods based on joint data training, and meta-learning approaches. Ensemble, for instance, refers to multiple methods that utilize pretrained decoding models to decode the EEG data of a new participant, and the result is obtained by integrating different classifier decisions through voting or other strategies. Nicholas et al. proposed an ensemble method based on Riemannian geometry and spectral transfer that is termed Spectral Transfer-learning using Information Geometry (STIG) [11]. This method employs spectral meta-learning to integrate existing classifiers for classifying new participants, thus achieving a balanced accuracy (BA) of 78% in the RSVP-BCI paradigm. Xiao et al. also developed a method called discriminative canonical pattern matching, which is another ensemble approach that is applicable to ZC classification in the RSVP paradigm [12].

Pooling is a simple and effective ZC method that uses data from different EEG datasets to train a single model, with the aim of enhancing the generalization performance across different individuals. In 2020, Lee et al. employed EEG data from 55 subjects in a P300 speller paradigm to jointly train a variant of the EEGNet model, which could be directly applied to decode EEG data from new individuals [13]. Several studies have focused on the unique features of BCI paradigms and their variability across different individuals, thus tailoring the design of the decoding model structures to enhance performance in ZC scenarios. Li et al. proposed a graph-node classification method based on convolutional neural networks (CNN) and adaptive graph learning for ZC decoding, established an adaptive connectivity graph for similar samples, and used a graph attention network to aggregate the features of similar samples, thus obtaining favorable results [14]. However, because of the need to construct graphs with multiple samples as the input, the actual model training time is relatively long. 

“Learning to learn”, which is a fundamental concept in meta-learning, closely resembles the learning mechanism employed by the human brain, which accumulates experience from various learning processes for future tasks [15]. The concept of meta-learning was initially applied in the field of computer vision recognition, specifically for the recognition of physiological images in scenarios including small sample sizes. Koch et al. introduced a meta-learning method based on a Siamese neural network, which calculated the L1 distance between the test images and anchor points as a metric and achieved an accuracy of 92% in image recognition in the Omniglot dataset [16]. Furthermore, Vinyals et al. proposed a meta-learning method that used a matching network, employed cosine distance as a measure, and implemented classification based on the probability distribution of test subject labels. This method achieved an accuracy of 93.8% on the Omniglot dataset [17]. In 2022, Wei et al. introduced a meta-learning-based prototype-matching network termed EPMN. The method started with the common features of EEG data [18]. By enabling the model to “learn to learn”, a feature prototype was extracted to capture common characteristics. Subsequently, the EEG data of new individuals were matched with these feature prototypes to achieve ZC training. The EPMN method achieved an average BA of 86.34% on a self-dataset of 31 participants. 

Inspired by the prototype network reported by Wei et al. [18], this paper continued the basic framework of the prototype network. It achieved classification in the RSVP paradigm by comparing the distance between the data in the test set and pre-established event-related potential (ERP) prototype features. During the model training phase, a unique feature prototype was generated for each category of support set samples. Subsequently, the category of a sample was determined by calculating the distance between the query set sample and the prototype in the classification phase. Such an approach, which requires only the extraction of prototypes from the network model, can afford powerful generalization capabilities. This method is particularly well-suited to high-cost EEG signals, including those in labeled paradigms, such as RSVP. In our study, an identical theoretical foundation was adopted as EPMN to explore a new high-performance ZC decoding algorithm based on RSVP-BCI. Considering the practical application of EPMN, the use of the Manor–CNN network as the classifier, which has fixed-scale receptive fields during feature extraction, constrained the ability of the model to extract crucial features [19]. This limitation resulted in a suboptimal classification performance. To address this drawback, a multiscale attention mechanism network was introduced for feature extraction to enhance model efficiency. To improve the generalization ability of the model, a hybrid attention mechanism was incorporated after feature extraction to enhance algorithm performance. Finally, suitable data augmentation and channel selection methods were integrated into the model.

## 2. Materials and Methods

### 2.1. Dataset 1

#### 2.1.1. Participants and Data Acquisition

**Figure 1:**
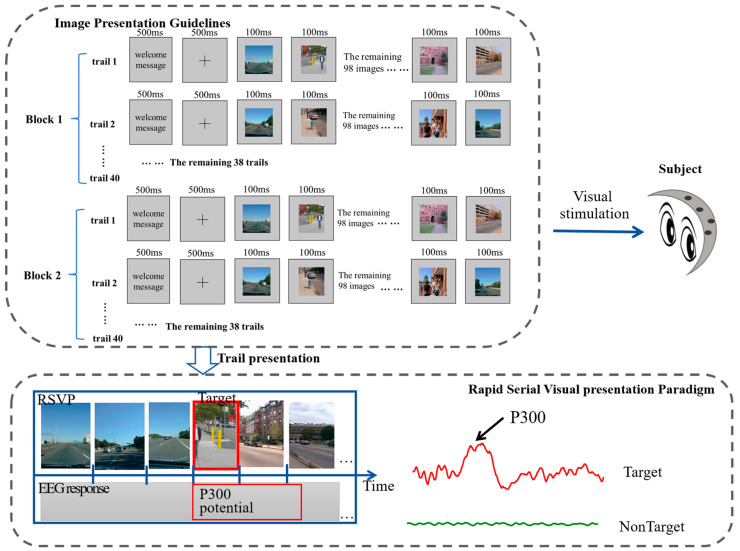
RSVP stimulus presentation design in Dataset 1.

One of the experimental datasets was derived from a publicly available RSVP dataset released by Zhang et al. in 2020 [20] (http://bci.med.tsinghua.edu.cn/, accessed on 1 December 2023). We refer to this dataset as Dataset 1 in the following content. This dataset comprises RSVP data from 64 individuals. The experimental setup consisted of two blocks, each consisting of 40 trials, as shown in Figure 1. Each trial included a 500 ms “focus” cue, followed by the presentation of 100 images, with 1–4 target images (including the image of a human) randomly embedded among them. The experiment was divided into two sessions, which were labeled “A” and “B”, respectively. The RSVP paradigm presented a street scene image in which the target image was the presence of people in the street scene, as opposed to the nontarget images, in which the background was devoid of people. The image presentation rate was set at 10 Hz, with the “ref” electrode placed at the vertex.

#### 2.1.2. Data Preprocess

To ensure that the impedance of the data of all participants remained below 10 kΩ, the “EOG1” and “EOG2” channels were removed from the raw data of the 64 channels. The removed EEG data underwent bandpass filtering using a 4th-order Butterworth filter in the frequency range of 2–30 Hz. In addition, a channel selection method based on a large-scale sparsity problem was considered to optimize multiple objectives and enhance the classification performance of the new model. Because of the imbalance in the target-to-nontarget ratio of the data collected here, this paper also addressed the issue of data imbalance in the preprocessing stage. We employed a data augmentation method based on a balanced generative adversarial network to further mitigate the effects of class imbalance.

### 2.2. Dataset 2

#### 2.2.1. Participants and Data Acquisition

Another RSVP dataset was derived from a self-collected dataset. This dataset included 9 experimental participants aged between 22 and 26 years, with an average age of 23.44 years, comprising 2 females and 7 males, all of whom were right-handed. None of them had previous experience with RSVP-BCI. Moreover, no participants had any history of visual impairments, neurological diseases, or injuries. All subjects had normal vision or vision corrected to normal, signed informed consent forms, and received financial compensation for their participation. The experiment was approved by the Review Committee of the Beijing Institute of Machinery and Equipment. We refer to this dataset as Dataset 2 in the following content.

**Figure 2:**
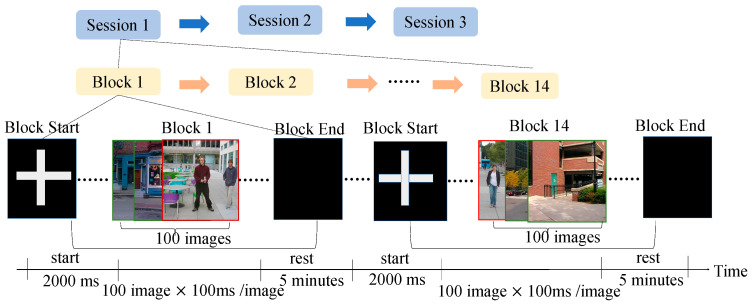
RSVP stimulus presentation design in Dataset 2.

The stimulus images used in the RSVP experiment were street view images collected from the Massachusetts Institute of Technology’s Computer Science and Artificial Intelligence Library. Participants were asked to identify target images from a randomly ordered sequence of images. The experimental design for data collection consisted of three phases. Each session included 14 blocks, with each block starting with a 2000 ms cross-mark to indicate the beginning of the experiment, followed by 100 images presented at a frequency of 10 Hz. The duration of each block was approximately 5 min, with a total of 4200 images presented throughout the session. The average rest time between two sessions was about 15 min to alleviate participant fatigue. Participants were required to identify target images (images showing humans, distinguished from nontarget images that did not contain humans) and count the number of target images. The ratio of target to nontarget images was 1:24. Figure 2 illustrates the framework of the RSVP paradigm.

#### 2.2.2. Data Preprocess

Here, the EEG recordings were captured using the Synamps2 system (Neuroscan, Inc., Brentwood, UK) at a sampling frequency of 1000 Hz. The EEG data were stored in the “.cnt” format, incorporating all 64 Ag/AgCl electrode channels arranged in alignment with the 10–20 system. The reference electrode was positioned at the left mastoid, and the impedance of the electrodes was kept under 10 kΩ. The system applied a filter to the EEG data ranging from 0.1 Hz to 100 Hz. Additionally, to eliminate interference from electrical power sources, the EEG data underwent filtering to exclude frequencies above 50 Hz. Two malfunctioning channels (M1 and M2), which exhibited electrode impedances exceeding 10 kΩ, were excluded from the analysis.

During the preprocessing stage, the continuous EEG recordings were subjected to a bandpass filter from 2–30 Hz using a 4th-order Butterworth filter. The data for each block were divided into EEG trials, with each trial being associated with a specific picture. In the experimental design of this study, each participant was presented with 14 blocks of EEG samples, with each block consisting of 100 trials, during a single session.

### 2.3. Network Architecture

**Figure 3:**
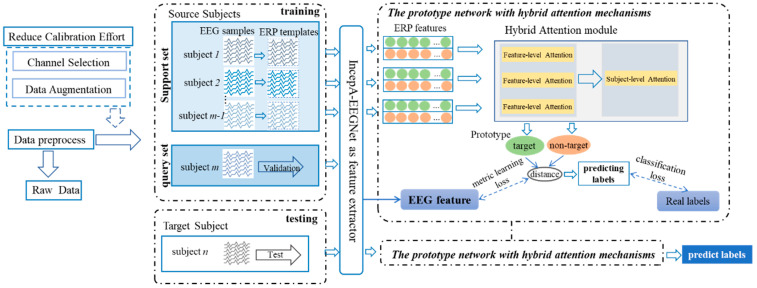
Overall framework of the Attention-ProNet method.

The Attention-ProNet framework integrated the core principles of matching networks from meta-learning, which were tailored with custom improvements for brain network features. The Attention-ProNet framework is illustrated in Figure 3. Initially, the raw data were trained, with one epoch including *m* meta-task training. In each meta-task, the m source domains were divided into a support set and a query set. The EEG sample from the support and query sets underwent a process that was capable of extracting low-dimensional features from different categories of EEG signals to reduce data dimensionality. Subsequently, a hybrid attention mechanism that encompassed EEG feature attention and subject-leave attention was employed to weight average low-dimensional EEG features and apply ERP prototypes, thus emphasizing crucial information within the brain networks [21]. Finally, the framework incorporated a matching network structure and applied the metric loss and classification loss to constrain the metric space of low-dimensional features. In the testing stage, classification was performed by mapping the distance between the EEG features and the ERP feature prototypes of each category to categorize EEG samples into matching prototypes of the nearest category. In summary, this framework was designed to enhance the understanding and processing of information within brain networks, particularly regarding EEG data, by leveraging the structure of the matching networks from meta-learning.

#### 2.3.1. Prototypes of ERPs

In RSVP tasks, there were similarities in the EEG characteristics among different subjects. Therefore, it was possible to learn specific prototypes for each category and use a prototype-matching network from prototype learning to obtain ERP prototypes.

Therefore, each ERP prototype was defined by Equation (1), where m is the number of subjects. This was similar to the hard-clustering algorithm.

(1) $$p^{k}=\frac{1}{m-1}\sum_{i=1}^{m-1}\;f\left(x_{i}|y_{i}=k\right),k\in \{0,1\}$$

where $x_{i}$ represents an EEG sample with c channels and t time points, $y_{i}$ represents the two fundamental classes of targets, *m* denotes the number of subjects, one of which is used for validation, and f represents the features extracted by the feature extractor. To avoid significant differences in features across subjects, the feature processor conducts batch normalization prior to output. This formula is used to extract the features of each subject and then calculate the average, for which $p^{k}$ serves as a common template. This means that the “target” and “nontarget” features of the subjects are extracted in this manner to identify their common features. Then, the test samples are matched with these two templates, that is, by calculating the minimum Euclidean distance. In this process, no test samples are involved in the training process.

#### 2.3.2. EEG Feature Extraction

The feature extraction technique of Attention-ProNet drew inspiration from our previously proposed IncepA-EEGNet [22]. It integrated attention mechanisms with a multiscale convolutional strategy to enhance the extraction of crucial information from P300 ERP signals. In the case of IncepA-EEGNet, the first layer combines multiscale convolution modules, deep convolution modules, and Squeeze-and-Excitation Attention (SE-Attention) [23]. The second layer encompasses deep convolution and efficient channel attention (ECA-Attention) [24], with ECA-Attention replacing the point convolution in EEGNet. Previously, this approach maximally extracted ERP features in RSVP, thus yielding favorable results in individual subjects. Given the need for efficient extraction of low-dimensional features, the classifier layer of the IncepA-EEGNet network was removed. The features obtained from the convolutional layers were mapped to a 1024-dimensional feature space, normalized, and then output.

#### 2.3.3. Feature-Level Attention Module

EPMN employs Euclidean distance to measure the distance between EEG features and ERP prototypes by assuming that the contribution of EEG features from participants is equal and neglecting useful information. Therefore, to extract informative portions from these features for classification, this study introduced a feature-attention mechanism to capture EEG features that were valuable for classification decisions. 

**Figure 4:**
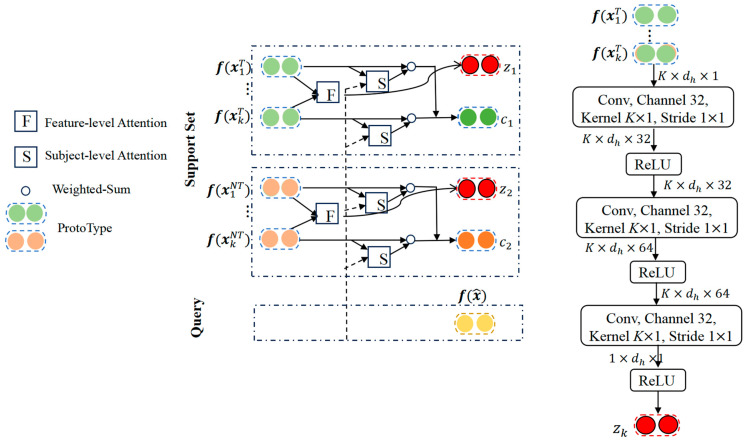
Architectures of hybrid attention mechanism.

Feature-level attention emphasized important dimensions in the feature space by adding weight information to the low-dimensional features of target and nontarget ERPs. As shown in Equation (2) and as shown in Figure 4 (right), the feature-attention mechanism acquires low-dimensional features of target and nontarget ERPs from the Incepa-EEGNet feature extractor. It then emphasizes important dimensions in the feature space by adding weight information. Here, $z_{k}$ represents the score vector obtained by the feature-level attention, with features $f\left(x_{1}^{T}\right),f\left(x_{2}^{T}\right),\ldots ,f\left(x_{n}^{T}\right)$ extracted from the feature extractor-Incepa-EEGNet, indicating the number of subjects in the support set. These features are input into the convolutional network and, using the ReLU activation function, feature-level probability weights for different categories are obtained, signifying the varying degrees of importance of low-dimensional features of ERP focused on by target and nontarget images.

(2) $$d\left(f\left(\hat{x}\right),p\right)=z_{k}\cdot {\left(f\left(\hat{x}\right)-p^{k}\right)}^{2}$$

where $z_{k}$ represents the attention score vector obtained through feature-level attention and k is the k-th category. The $f\left(\hat{x}\right)$ of each feature were obtained from IncepA-EEGNet, with $\hat{x}$ representing the ERP features of the unknown subject and $p^{k}$ representing the ERP prototypes. By taking the difference between the unknown subject and the matching template and multiplying it by the subject’s attention score, we can find the maximum probability value for the matching sample that is closest to the unknown subject. This approach resembles a subject selection method.

#### 2.3.4. Subject-Level Attention Module

Different signals from participants may have varying effects on the results during the generation of the ERP prototypes. This implies that using the same weight for processing all participant data may result in a significant bias, especially when the EEG signals of a specific participant differ markedly from those of others or when noise is more pronounced in the data. It is essential to introduce an attention mechanism at the sample level to mitigate bias issues. This is analogous to source-domain-selection strategies, which place more attention on subjects whose low-dimensional ERP features are relevant to the query set.

We argue that not all subjects from support are equal when given a query, and with each subject representation being given a weight, the subject-level attention can be represented by Equations (3)–(5).

(3) $$e_{k}=sum\{\sigma (g(f(x_{k})⊙g(f(\hat{x}))))\}$$

$x_{k}$ represents the feature vector of the support set subject sample and $\hat{x}$ from the query set subject sample, and g(.) denotes a linear transformation followed by element-wise product operations between $f(x_{k})$ and $f(\hat{x})$, where σ(·) is the activation function, tanh is chosen for σ(·) to map the element-wise product result to range [–1,1], and sum(.) indicates the summation of all elements in a vector.

(4) $$\alpha_{i}=\frac{\exp(e_{i})}{\sum_{k=1}^{N-1}\exp(e_{k})}$$

Here, $\alpha_{i}$ was obtained through the Softmax function (using $e_{i}$ and $e_{k}$ as the corresponding parameters).

(5) $$p^{k}=\frac{1}{m-1}\sum_{i=1}^{m-1}\alpha_{i}\;f\left(x_{i}|y_{i}=k\right),k\in \{0,1\}$$

Therefore, we replace Formula (1) with Formula (5), with subject-level attention assigned different weights to the corresponding subjects. Subjects closer to the query set were given higher weights, thus increasing the influence of support set subjects and thereby improving classification performance. Compared with the low-dimensional feature vectors of the original average subjects, the Attention-ProNet proposed in this paper was more robust.

### 2.4. Loss Function Definition

The loss function in this study was divided into two components: metric loss and prediction loss. For the metric loss function, the predicted probability of the label y=k for each sample x was calculated according to Equation (6).

(6) $$p\left(y=k|\hat{x}\right)=\sum_{y^{k}\in \left\{target,Nontarget\right\}}\lambda d(\hat{x},p)y^{k}$$

The prediction loss function primarily involved predicting the probabilities of two categories by employing the distance Softmax function, as shown in Equation (7), where d represents the Euclidean distance.

(7) $$\lambda \left(d\left(\hat{x},p\right)\right)=\frac{\exp(d^{k}(f\left(\hat{x}\right),p))}{\sum_{j}{\exp(d}^{j}(f\left(\hat{x}\right),p))}$$

The classification prediction loss function for the prototype network was represented by the cross-entropy loss, as shown in Equation (8).

(8) $$L_{class\_loss}=-\frac{1}{N}\sum_{i=1}^{N}\left[y_{i}logP\left(y_{i}|\hat{x}\right)+(1-y_{i})logP\left(y_{i}|\hat{x}\right)\right]$$

The metric loss refers to the computation of the distance between an unknown sample and the prototype category. If the unknown sample was closer to the ERP prototypes of the category “target”, then it was considered to belong to the “target” category, and vice versa. The prototype network metric loss was defined in Equation (9), where the distance between the unknown subject and the ERP prototypes of different categories was computed by incorporating an attention mechanism. The design of $L_{metric\_loss}$ adhered to the concept of minimizing the distance within categories while maximizing the distance between categories, as articulated in Equation (10).

(9) $$L_{metric\_loss}=\sum_{y=k}d\left(f\left(\hat{x}\right),p\right)-\sum_{y\neq k}d\left(f\left(\hat{x}\right),p\right)$$

(10) $$\underset{\theta }{\min}\left|L_{class\_loss}+L_{metric\_loss}\right|$$

### 2.5. Meta-Training

**Figure 5:**
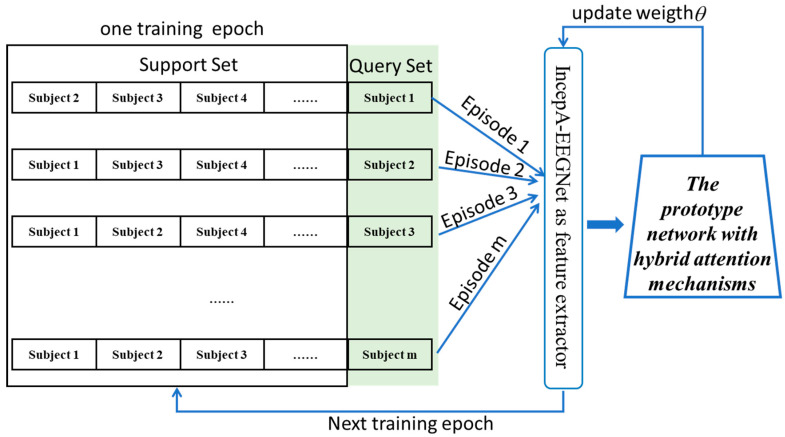
The meta-learning training process.

To prevent contamination of the query set EEG samples during the training process, a meta-learning training process was used [25,26], as illustrated in Figure 5. The model was trained through multiple epochs. During each epoch, all participants in the training set were iterated individually to update the model. Within an epoch, training was conducted episodically, with each epoch comprising m episodes. In one episode, each subject from the support set was alternately used as the query set, whereas the data from the remaining subjects constituted the training set. In one epoch, all samples from the training set were sequentially used for validation, and EEG samples were input into the Attention-ProNet network after obtaining the ERP features to complete one training iteration.

### 2.6. Evaluation Metrics

This study employed the commonly used balanced accuracy (*BA*) item as the classification evaluation metric in the comprehensive RSVP classification index. The formula for calculating *BA* is as shown in Equation (11). *BA* is from 0 to 1, where 0 is the worst possible performance and 1 is the best possible performance.

(11) $$balance\;accuracy(BA)=\frac{1}{2}(\frac{TN}{TN+FP}+\frac{TP}{TP+FN})$$

(12) $$TPR\;(True\;Positive\;Rate)=\frac{TP}{TP+FN}$$

(13) $$TPR\;(True\;Negative\;Rate)=\frac{TN}{TN+FP}$$

Here, *TP* represents the number of correctly predicted targets among the identified target instances, *FP* is the count of incorrectly predicted target instances, *FN* denotes the number of nontarget instances that were misclassified as targets, and *TN* represents the count of correctly identified nontarget instances. We also compared the method using the area under the curve (AUC). The Wilcoxon tests were utilized to assess the differences between groups, incorporating the statistical concept of the *p*-value. This study conducted two-tailed significance tests, where a *p*-value less than 0.05 indicated statistically significant differences.

## 3. Results

### 3.1. Experimental Setting

The feature extraction module, IncepA-EEGNet, employed in this study used the Adam optimizer with a learning rate of 1 × 10^−4^ and a learning momentum of 0.9. The size of the query set was set to 12. To prevent overfitting, a cosine annealing learning rate was applied to gradually decrease the learning rate. The experiments were carried out in an environment with a 24-core CPU and a GPU equipped with a 3090Ti with a CPU memory of 192 GB. The code was implemented using the Python-PyTorch framework. The experimental environment used for subsequent sections remained consistent with this setup. Here, the source domain (training set) EEG data were divided into two subsets, a query set and a support set, with a ratio of 1:62. The remaining subset was reserved for testing. During each iteration, the EEG data from the source domain were alternately designated as the query set, whereas the remaining EEG data served as the support set.

### 3.2. Comparable Experiment Networks

The ZC method proposed here, Attention-ProNet, afforded new subject target and nontarget classification tasks through representation learning by leveraging the mapping relationship between unknown EEG data and the ERP stimulus data. In this paper, Attention-ProNet was compared with other known RSVP ZC methods from the literature, including STIG [11], HDCA [27], EEGNet [28], and EPMN [18].

We compared these four models with our Attention-ProNet tool, as follows.

**STIG:** Proposed spectral transfer method based on information geometry. This method utilizes a minimum distance Riemannian mean classifier to integrate ZC across training data from multiple participants. It combines and arranges decisions from different classifiers.

**ZC-HDCA:** HDCA is the baseline algorithm for RSVP target detection. It achieves ZC using pooling methods, and generalizes the model’s ability to extract known subject EEG features using EEG data from multiple subjects. This enables the model to classify unknown subjects.

**ZC-EEGNet:** EEGNet is a multilayer CNN that includes deep convolutional layers and separable convolutional layers. This network is employed for feature extraction in various BCI paradigms and allows the use of pooling methods for training the EEGNet network.

**EPMN:** The framework of the EPMN model is described as follows. During the model training phase, this study introduces a meta-training strategy that divides the source domain into a support set and a query set. The model takes as paired inputs the EEG samples from the query set and the ERP templates from the support set, with the feature extractor adopting the network model proposed by Manor et al. [19], mapping the inputs to a metric space. The ERP prototype-matching module constrains the metric space with a classification loss function and the proposed metric learning loss function. In the testing phase, the ERP templates from the source domain and the test EEG samples are jointly input into the model, and classification is achieved by calculating the distance in the metric space between the EEG samples of the test set and each class of ERP prototypes; the EEG samples are classified into the category of the nearest ERP prototype, thereby achieving zero-shot learning.

**Figure 6:**
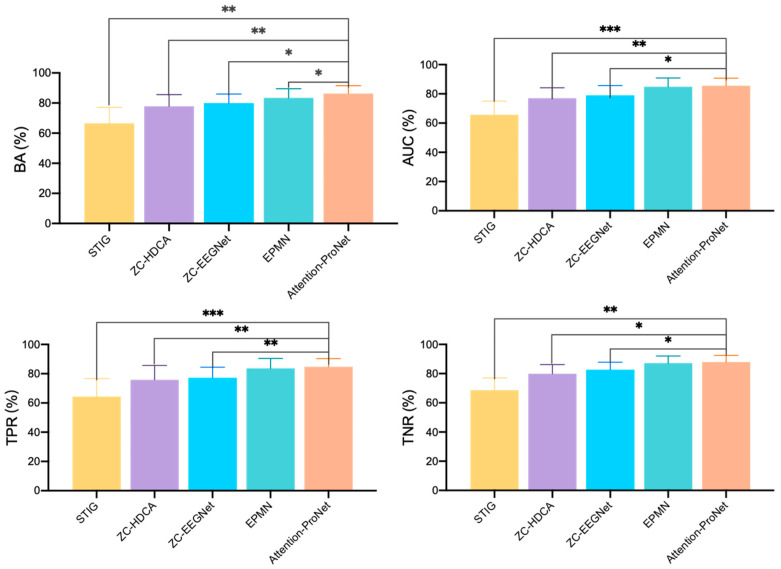
The compared results of different ZC methods in Dataset 1. *: *p*-value < 0.05, **: *p*-value < 0.01, ***: *p*-value < 0.001.

**Figure 7:**
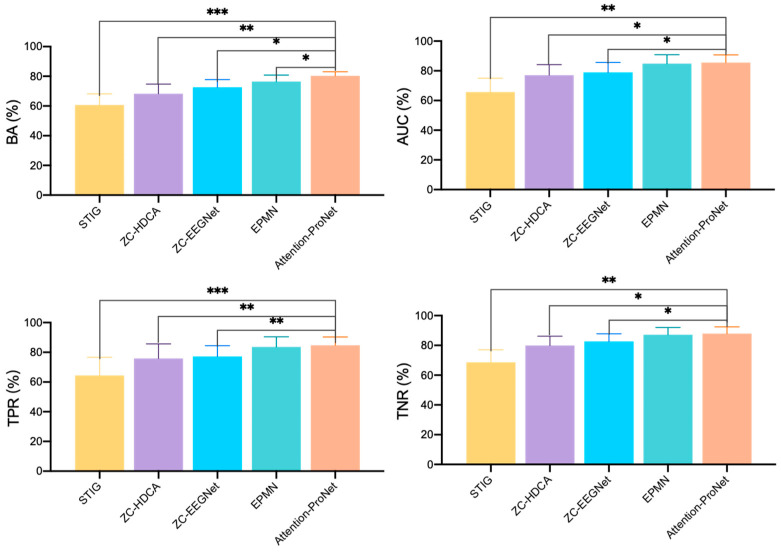
The compared results of different ZC methods in Dataset 2. *: *p*-value < 0.05, **: *p*-value < 0.01, ***: *p*-value < 0.001.

As shown in Figure 6 and Figure 7, the comparison reveals that in target recognition tasks, Attention-ProNet achieves higher BA, AUC, TPR, and TNR, followed by EPMN. The remaining two pooled ZC methods are ZC-HDCA and ZC-EEGNet. STIG, which employs an ensemble approach, is relatively the poorest among the five methods, with various evaluation metrics around only 60%. Compared with the proposed Attention-ProNet method, EPMN exhibits a significant difference in BA among the ZC methods, and although the other three evaluation metrics are slightly lower than those of Attention-ProNet, they do not show a significant difference. This suggests that the use of IncepA-EEGNet as a feature extractor, which integrates attention mechanisms and multiscale convolution strategies, and the incorporation of attention mechanisms at both the feature and subject levels, yield a stronger capability for future subject adaptability compared with EPMN.

### 3.3. Results of the Source-Domain-Selection Experiment

**Figure 8:**
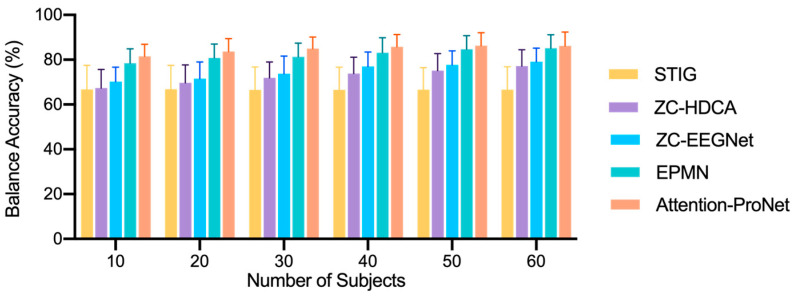
Results of the source-domain-selection experiment.

Figure 8 illustrates the conclusions obtained for participant cohorts of 10, 20, 30, 40, 50, and 60 individuals. As the number of participants in the support set increased by increments of 10, our proposed method maintained a BA of over 85% after reaching a support set size of 40–60 participants, affording an improvement of 2.6% compared with EPMN. This improvement may be attributed to the enhanced generalization of unknown subject data resulting from improved feature extraction. However, beyond 40 participants, all four ZC methods exhibited no significant improvement in BA. This might be because Attention-ProNet may experience saturation in extracting EEG information when the participant count exceeds 40 individuals.

However, ZC-HDCA and ZC-EEGNet yielded a noticeable increase in BA beyond 40 subjects. The BA values at 60 subjects were 67.17% ± 7.27% and 79.11% ± 8.03%, respectively. Conversely, STIG required a lower number of subjects and achieved a higher accuracy at around 20 subjects. These results suggest that the number of subjects affected the ZC outcomes. As the number of subjects increased, the performance of the meta-learning methods remained relatively stable. Therefore, in ZC experiments based on meta-learning, the number of subjects should be carefully considered to identify the size of the EEG cohort that is most suitable for the RSVP task.

### 3.4. Ablation Study

To assess the impact of individual modules on the performance of the Attention-ProNet network, we performed ablation experiments using ProNet, IncepA-EEGNet, subject specific attention, and feature-attention mechanisms.

As indicated in Table 1, there were significant differences in the comparative patterns of this method (*p*-value < 0.05). Comparative patterns A and B vs. pattern E exhibited even more significant differences (*p* < 0.01). Comparative patterns C and D vs. pattern E also showed significant differences (*p* < 0.01). From the results presented in the table, it was evident that the subject attention mechanism, as opposed to the feature-attention mechanism, resulted in a higher BA for pattern D vs. pattern C. This suggests that the subject attention mechanism contributes to Attention-ProNet to a greater extent. For RSVP, the impact of different feature-attention mechanisms on ERP prototypes is more crucial than the attention mechanisms of different subject examples. Therefore, achieving element-wise multiplication of low-dimensional features from ERP helped achieve a superior classification performance.

## 4. Discussion

In this study, we proposed a novel ZC method, Attention-ProNet, which integrated various attention mechanisms for cross-modal target recognition tasks in the RSVP paradigm. The series of experiments reported here confirmed that our model exhibited a favorable classification performance on an open dataset. The ablative experiment confirmed the crucial impact of feature-attention mechanisms on ERP prototypes. To assess comprehensively the performance of the proposed network, we conducted further experiments and discussions pertaining to cross-day evaluation, channel selection, data augmentation, and other aspects, thus building upon the preliminary experimental results and our prior research foundation.

### 4.1. Cross-Day Performance

The current research on EEG signals focuses predominantly on single trials, with the EEG data collected for training and testing often concentrated within the same period. However, cross-temporal studies are scarce. Because EEG signals may exhibit variations in brain activity at different times, this study aimed to assess the cross-temporal capabilities of Attention-ProNet. This research used a dataset that is openly available from Li et al. for ZC testing across days [29] (https://doi.org/10.6084/m9.figshare.12824771.v1, accessed on 1 January 2024). The dataset comprised EEG data from 14 healthy subjects, with a 23-day interval between two data collection sessions from each subject. Each experiment encompassed 4200 images, including 56 target images. Here, the data from the first experiment were used as the training dataset, whereas the data from the second experiment were used as the testing dataset. Image preprocessing was performed as described in Section 2.2. 

**Figure 9:**
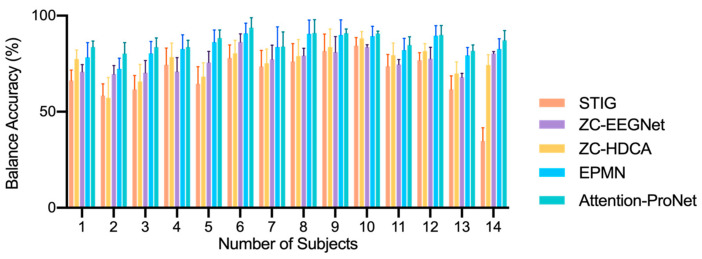
The cross-day classification comparison of different ZC networks.

As shown in Figure 9, the algorithm proposed in this paper, Attention-ProNet, was compared with the STIG, HDCA, EEGNet, and EPMN algorithms. The average BA of Attention-ProNet was 86.14%. Compared with the HDCA method, the average BA was improved by 10.33%. In turn, compared with the SIM (Signal-to-noise ratio Maximizer, which could maximize the SNR of ERPs) + HDCA method, the average BA was increased by 8.3% [30], and, compared with the EPMN tool, the average BA was improved by 2.18%. This indicates that the algorithm proposed in this paper afforded good robustness in cross-temporal experiments, effectively extracted common features of ERPs, and was not affected by the physiological rhythms of cross-temporal EEG signals.

### 4.2. Channel Selection

To enhance the performance of the algorithm further, this study incorporated the channel selection module and data augmentation methods proposed by the research team in earlier work by Xu et al. into the preprocessing stage of the classification model [31,32]. The channel selection module uses the SparseEA algorithm mentioned above, which is a multi-objective channel selection approach that is based on large-scale sparse evolution. We added multiple operations to adapt the algorithm to channel selection schemes for brain–computer interfaces. The RSVP Benchmark dataset proposed by Zhang et al. was employed as the validation dataset, and leave-one-out cross-validation was used for experimentation. The ZC performance of the STIG, HDCA, EEGNet, EPMN, and Attention-ProNet algorithms in channel selection was compared in the experiments, and the results are presented in Table 2.

According to Table 2, it was evident that after applying the channel selection scheme, the accuracy of the EEG signals of the subjects was improved compared with that obtained when the channel selection module was not included. These two datasets exhibit the same trend, but the values of Dataset 1 are higher than those of Dataset 2. This may be due to Dataset 1 having a larger volume of data, resulting in better generalizability. The most significant improvement in BA was observed in the STIG and ZC-HDCA cases. This was mainly because our channel selection optimization scheme was based on traditional classifier principles. Therefore, the STIG and ZC-HDCA networks might inherently be more compatible with this channel selection approach, leading to the most significant enhancement in BA. Close behind, the EPMN model demonstrated notable improvements, with a 1.66% increase in Dataset 1 and a 1.49% rise in Dataset 2 following channel selection optimization. In comparison, the newly proposed Attention-ProNet method showed a relatively modest improvement in BA, particularly in Dataset 1, where it only saw a 0.94% increase, accompanied by a *p*-value of 0.0367. This analysis suggests that the method proposed here employs multiple attention mechanisms similar to filters in both spatial and temporal domains. To a certain extent, this optimized the weights of the EEG data for different channels, leading to a limited improvement space.

### 4.3. Data Augmentation

The Class Imbalance Problem has been a consistently significant hindrance to the classification performance of the RSVP paradigm. To alleviate the imbalance issue, our research team previously proposed a BWGAN-GP network. This network, which combines GAN and autoencoder, is capable of generating high-quality EEG signals for the minority class. Here, the data generated by this network were incorporated into the proposed ZC model, and experiments were conducted on a balanced dataset that included both artificial EEG data and real data. The results are presented in Table 3.

From Table 3, it is evident that the different algorithms afforded an improvement in BA after data augmentation. After data augmentation, the classification performance of several methods improved. The increases were relatively smaller for the machine-learning-based STIG and ZC-HDCA. Among the deep learning algorithms, the classification performance of ZC-EEGNet increased by 2.53% in Dataset 1 and by 1.52% in Dataset 2. The improvements in classification performance were relatively more significant for EPMN and Attention-ProNet, especially in Dataset 1, where they increased by 1.86% and 1.99%, respectively. This indicates that data augmentation can enhance the classification performance of ZC algorithms. The primary reason for this improvement is the increased diversity of the EEG samples, which enhances the performance and robustness of the classifier in ZC problems. Furthermore, the experiment compared the performance of channel selection and data augmentation operations simultaneously applied to different classifiers. Notably, Attention-ProNet exhibited a significant improvement compared with EPMN, with a classification accuracy of 88.65% ± 4.66% in Dataset 1.

### 4.4. Limitations

The computational complexity of our proposed Attention-ProNet is evaluated based on the number of model parameters and the training and inference time of the model. For comparison purposes, Table 4 presents the average BA, model parameter count, training time, and inference time of Attention-ProNet and its competitors on two datasets. It is worth noting that the time consumption of all models was measured on a hardware platform equipped with an Intel E5-2620 v3 CPU 192G and an NVIDIA GTX 3090Ti GPU. The software environment includes Python 3.10, PyTorch 1.16.1, and CUDA 12.0. From Table 4, it can be observed that EEGNet has the lowest computational complexity. However, due to its simple network architecture, it struggles to effectively capture the most discriminative features in EEG signals, resulting in a lower average BA. However, compared to EPMN, Attention-ProNet has more parameters and requires more time due to the integration of the attention mechanism, which is a limitation of our algorithm.

This paper does not discuss the robust boundary issues of the attention mechanism, such as the impact on the prototype network caused by not removing signals with larger amplitudes, like electromyography and eye movement noise, during the collection and preprocessing of EEG signals. Additionally, if the data collection from a particular subject is abnormal, it will also affect the prediction accuracy of the matching template. These issues will be explored in our future research.

## 5. Conclusions

This study introduced a prototype-matching network, Attention-ProNet, that integrated multiple attention mechanisms for ZC in RSVP tasks. The proposed method used a prototype network to acquire ERP prototypes and employed Attention-ProNet to classify the distances between different ERP prototypes and EEG signals from unknown subjects. Building upon the prototype network, IncepA-EEGNet was introduced to extract low-dimensional features from the EEG signals of the subjects. Feature-attention mechanisms and subject-sample-attention mechanisms were designed to enhance the weights of useful features among the low-dimensional features of different subjects, thereby improving the generalization ability of the model. The experimental results demonstrated that the proposed Attention-ProNet outperformed the current ZC methods in the RSVP paradigm. Finally, the integration of channel selection and data augmentation modules further improved the performance of the model in the RSVP ZC classification problem. This provides a reliable direction for future RSVP-BCI ZC research and supports the practical application of RSVP-BCI online systems.

## Figures and Tables

**Table 1:** Ablation study in the proposed method with various network patterns. ✓ and × indicate whether this is the corresponding part.

Model	Without the ECA Attention Mechanism, IncepA-EEGNet	IncepA-EEGNet	Feature-Level Attention	Subject-Level Attention	BA (%) Mean ± Std	*p*-Value
A	✓	×	×	×	81.22 ± 7.86	**
B	✓	✓	×	×	85.78 ± 6.26	**
C	×	✓	✓	×	85.90 ± 6.12	*
D	×	✓	×	✓	86.14 ± 5.91	*
E	×	✓	✓	✓	86.33 ± 5.18	-

‘*’ represents a significant difference in BA before and after channel selection, *: *p*-value < 0.05, **: *p*-value < 0.01.

**Table 2:** The average results of BA in ZC models with channel selection.

Model	Dataset	Before Channel Selection (%)	After Channel Selection (%)
STIG	Dataset 1	66.89 ± 10.64	69.25 ± 9.51 **
	Dataset 2	60.67 ± 8.52	62.75 ± 7.77 **
ZC-HDCA	Dataset 1	77.92 ± 7.65	81.43 ± 6.61 **
	Dataset 2	68.23 ± 6.56	70.65 ± 6.43 **
ZC-EEGNet	Dataset 1	79.87 ± 6.19	81.00 ± 5.85 *
	Dataset 2	72.54 ± 5.22	74.12 ± 5.07 *
EPMN	Dataset 1	85.42 ± 6.10	87.08 ± 4.21 *
	Dataset 2	76.43± 4.44	77.92 ± 4.19 *
Attention-ProNet	Dataset 1	86.33 ± 5.18	87.27 ± 5.56 *
	Dataset 2	80.29 ± 2.79	82.15 ± 2.38 *

‘*’ represents a significant difference in BA before and after channel selection, *: *p*-value < 0.05, **: *p*-value < 0.01.

**Table 3:** The average results of BA in ZC models with data augmentation.

Model	Dataset	Before Data Augmentation (%)	After Data Augmentation (%)	Before Data Augmentation and Channel Selection (%)
STIG	Dataset 1	66.89 ± 10.64	67.50 ± 10.21 *	70.11 ± 8.79 ^+++^
	Dataset 2	60.67 ± 8.52	61.34 ± 9.88 *	63.85 ± 9.88 ^+++^
ZC-HDCA	Dataset 1	77.92 ± 7.65	78.16 ± 4.98 *	80.67 ± 5.23 ^++^
	Dataset 2	68.23 ± 6.56	69.81 ± 5.34 *	70.67 ± 5.02 ^++^
ZC-EEGNet	Dataset 1	79.87 ± 6.19	83.90 ± 4.82 **	84.88 ± 4.45 ^++^
	Dataset 2	72.54 ± 5.22	74.06 ±3.65 **	75.75 ± 3.43 ^++^
EPMN	Dataset 1	85.42 ± 6.10	86.19 ± 4.77 **	87.20 ± 4.79 ^+^
	Dataset 2	76.43 ± 4.44	77.38 ± 3.72 **	79.20 ± 3.09 ^+^
Attention-ProNet	Dataset 1	86.33 ± 5.18	87.40 ± 4.94 **	88.65 ± 4.66
	Dataset 2	80.29 ± 2.79	81.23 ± 2.61 **	82.41 ± 1.89

‘*’ represents a significant difference in BA before and after data augmentation or data augmentation and channel selection, *: *p*-value < 0.05, **: *p*-value < 0.01. ‘+’ indicates the significant performance of different classifiers for Attention-ProNet under the simultaneous influence of data augmentation and channel selection operations, ^+^: *p*-value < 0.05, ^++^: *p*-value < 0.01, ^+++^: *p*-value < 0.001.

**Table 4:** Model parameters and computation times of different methods.

Model	Dataset	BA Mean (%)	Parameters (1 × 10^3^)	Training Time (s)	Testing Time (s)
STIG	Dataset 1	66.52	-	5.23	1.59
	Dataset 2	60.67		9.41	1.44
ZC-HDCA	Dataset 1	77.92	-	139.32	2.38
	Dataset 2	68.23		74.37	1.85
ZC-EEGNet	Dataset 1	79.87	4.07	123.54	0.19
	Dataset 2	72.54		72.89	0.07
EPMN	Dataset 1	85.42	72.50	19.34	0.35
	Dataset 2	76.43		7.41	0.22
Attention-ProNet	Dataset 1	86.33	141.23	28.59	0.39
	Dataset 2	80.29		12.54	0.26

## Data Availability

The data presented in this study are openly available at the following URL/DOI: http://bci.med.tsinghua.edu.cn/ and https://doi.org/10.6084/m9.figshare.12824771.v1, accessed on 13 September 2023.
